# Serum sclerostin levels in osteoporotic fracture patients

**DOI:** 10.1007/s00068-022-02017-7

**Published:** 2022-06-16

**Authors:** Erwin A. Gorter, Casper R. Reinders, Pieta Krijnen, Natasha M. Appelman-Dijkstra, Inger B. Schipper

**Affiliations:** 1 Departments of Trauma Surgery, Leiden University Medical Center, postzone K6-R, P.O. Box 9600, 2300 RC Leiden, The Netherlands; 2 Internal Medicine, Center for Bone Quality Leiden, P.O. Box 9600, 2300 RC Leiden, The Netherlands

**Keywords:** Sclerostin, Fracture, Osteoporosis, Bone mineral density, Biomarker

## Abstract

**Purpose:**

Sclerostin inhibits bone formation and stimulates bone resorption. Previous studies found a positive association between bone density and serum sclerostin, but literature on sclerostin levels in osteoporotic fracture patients is scarce. The aim of the present study was to compare the serum sclerostin levels in osteoporotic and non-osteoporotic fracture patients and to assess the correlation of the sclerostin levels with bone mineral density and vitamin D status.

**Methods:**

In this cross-sectional study, we included patients over 50 years, with an extremity fracture after low-energy trauma treated between 2012 and 2018, with biobank samples and available bone density measurements by Dual X-ray Absorption. Osteoporosis was diagnosed according the World Health Organisation criteria. Vitamin D deficiency was defined as a 25(OH)D concentration < 30 nmol/L. After defrosting biobank samples, serum sclerostin was measured using the human SOST (sclerostin) enzyme-linked immunosorbent assay kit. We prespecified a subgroup analysis including only female patients.

**Results:**

179 patients were included of whom 139(78%) were female. In 46 patients (25.7%), osteoporosis was diagnosed. Bone mineral density was positively associated with sclerostin levels (*r* = 0.17, *p* = 0.026) and patients with osteoporosis had a significantly lower serum sclerostin compared to non-osteoporotic fracture patients (mean 41.9 pmol/L vs 48.1 pmol/L; *p* = 0.03). This difference remained significant after correction for potential confounders. Similar results were found in the subgroup of female patients. No association between serum sclerostin and vitamin D deficiency was found.

**Conclusion:**

Osteoporotic fracture patients had lower levels of sclerostin than non-osteoporotic fracture patients. Future research should focus on the use of sclerostin as biomarker for osteoporosis in fracture patients.

## Introduction

Sclerostin is expressed by the SOST gene and was originally identified in patients with sclerosteosis and van Buchem’s disease [1]. The genetic expression is suppressed by mechanical loading of osteocytes and is increased in case of unloading [2]. Sclerostin inhibits bone formation and stimulates bone resorption [3].

Many aspects of the effect range of sclerostin are still unclear. On one hand, an increased sclerostin concentration was found in fracture hematomas of trauma patients [4], suggesting a potential role for sclerostin during the fracture healing process. This possible involvement is supported by the finding that treatment with sclerostin antibodies enhanced bone formation in animal fracture models [5–8]. These findings have been confirmed by clinical studies on sclerostin antibodies. Antibodies used as anti-osteoporosis treatment showed increased bone density, bone formation and bone resorption-suppressing effects, [9] resulting in increased bone mass, strength and reduced fracture risks [10]. On the other hand, several previous studies discussed a positive association between bone density and serum sclerostin [11–27]. A limited number of studies investigated the relation between osteoporosis and sclerostin levels and found divergent results [28–32]. Three studies found a lower serum sclerostin concentration in patients with osteoporosis compared to non-osteoporotic patients [28, 29, 31]. One found no difference [30] and another study found an even higher level of sclerostin in osteoporotic patients [32].

Vitamin D, together with parathyroid hormone and fibroblast growth factor 23 (FGF-23), regulates the calcium and phosphate homeostasis and, like sclerostin, seems to be involved in the process of fracture healing [33]. In mice, deletion of the SOST gene resulted in a higher levels of serum 1α,25-dihydroxyvitamin D and lower levels of 24,25-dihydroxyvitamin D, possibly mediated by decreasing FGF-23 [34]. Clinical studies showed an inversed relationship between sclerostin and serum 25OHD concentration, whereas vitamin D supplementation paradoxically increased sclerostin levels [35–37]. On the other side, several studies have reported significantly lower levels of sclerostin after receiving vitamin D supplementation[37–40], or no effect at all [41–43]. This demonstrates that the exact interaction between vitamin D and sclerostin remains unexplained [3, 39, 43].

This study aimed to solve another piece of the puzzle regarding the relation between sclerostin and bone mineral density by comparing the serum sclerostin levels in a general fracture population of osteoporotic and non-osteoporotic patients with extremity fractures. We hypothesized that sclerostin levels would be positively associated with bone mineral density and subsequently lower levels of sclerostin would be found in osteoporotic fracture patients. Secondary, the relation between serum sclerostin levels and serum vitamin D status was studied in these patients.

## Materials and methods 

### Study design and patient population

This retrospective cross-sectional study included samples from two existing biobanks from the Leiden University Medical Center (LUMC). One was the Biobank for Bone and Mineral Disorders, the other the vitamin D-study biobank. The biobank for Bone and Mineral Disorders holds blood samples of various patient groups, including fracture patients, whereas the vitamin D-study biobank includes blood samples of adult fracture patients that had participated in a previous study on vitamin D and fractures [44].

We included patients over 50 years with an extremity fracture after a low-energy trauma, who had a dual emission X-ray absorptiometry (DXA) within 1-year post-fracture. No hip fracture patients were included, because hip fracture patients are not treated in our university hospital. Patients with pathological fractures or multi-trauma patients were excluded. For our fracture liaison service, patients are identified on the departments of orthopaedic surgery, trauma surgery and the emergency department. All fracture patients over 50 years are voluntarily invited, with the exception of patients with an isolated fractures of the skull, hands or feet, a life expectancy < 12 months or who are treated for osteoporosis elsewhere. Full workup consists of a anamnesis, full physical examination, DXA/VFA and laboratory screening and determination of secondary causes of osteoporosis and gonadal status, as described by Malgo et al*.* [45].

This study was approved by the medical ethics committee of the LUMC (B19.063). Previously, the medical ethics committee had approved the biobanking protocols for the Biobank for Bone and Mineral Disorders (EZ-BOT 2019-01) and the Vitamin D study (P12.058). The included patients for these studies gave written informed consent for the use of their blood and data in future studies at the time of blood sample collection.

### Bone mineral density

Bone mineral density (BMD) was measured at the lumbar spine (L1–L4) and at the left and right femoral neck using DXA with a Hologic QDR4500 scanner (Holologic, Bedford, MA, USA). T-scores were calculated using reference values from the Third National Health and Nutrition Survey (NHANES III) comparable with those of the Dutch population. Using World Health Organisation criteria, osteoporosis was defined as a BMD ≥ 2.5 standard deviation (SD) below the average value for young healthy women, expressed as a T-score of ≤ − 2.5. Osteopenia was defined as a BMD > 1SD but < 2.5 SD below the young adult mean (T-score between − 2.5 and − 1) and normal BMD was defined as a T-score ≥  − 1.0.

### Laboratory

Serum samples were collected and stored according to the institutional biobank protocol at – 80 ℃. After defrosting, serum sclerostin was measured using the human SOST (sclerostin) enzyme-linked immunosorbent assay kit (MSD K151HGC-2, Maryland, USA) in batch analyses with control samples used in all batches, with normal values based on healthy controls 40.0 pg/ml; (95% CI 37.2–42.9 pg/m) [46] as a reference.

Laboratory measurements were already performed at time of blood withdrawal. Serum calcium (albumin-corrected) and creatinine were measured by semiautomated techniques. Plasma intact parathyroid hormone (PTH) was measured by immulite 2500 (Siemens Diagnostics, Breda, The Netherlands) and serum 25-hydroxyvitamin D (25-OH D) by the 25-OH-vitamin D Total assay (DiaSorin D.A./N.V., Brussels, Belgium). Vitamin D deficiency was defined as a serum 25(OH)D concentration less than 30 nmol/L [47].

### Data

Data were collected from the electronic medical records of the included patients. This data included sex, age, length, weight, medical history (hypertension, diabetes mellitus, chronic obstructive pulmonary disease, hyper(para)thyroidism, renal insufficiency and previous fractures), use of medication (vitamin D supplementation, corticosteroids) at the time of fracture and intoxications as well as fracture location. Lab results of the serum vitamin D, calcium, PTH levels and estimated glomerular filtration rate (eGFR) were collected.

### Statistical analysis

All analyses were performed for the total patient group as well as for the subgroup of female patients. Patient groups were compared using the Chi-squared test for categorical variables, or the Fisher’s exact test (if expected cell counts < 5). Continuous variables were compared with a t test. A result was considered statistically significant when a *p* value of < 0.05 was found. The association between sclerostin levels and BMD was assessed by a linear regression analysis. A multivariate linear regression analysis was performed to evaluate the association between sclerostin levels and osteoporosis while correcting for potential confounders pointed out by the univariable analysis (*p* < 0.10). Also, a multivariate linear regression analysis was performed to evaluate the association between sclerostin levels and vitamin D deficiency. The Statistical Package for the Social Sciences (SPSS 23; IBM company, New York) was used for data analysis.

## Results

### Demographics

A total of 179 patients (24 from the Biobank for Bone and Mineral Disorders and 155 from the Vitamin D biobank) with 181 fractures were included, with a mean age of 65.1 years [standard deviation (SD) 10.5]. All 139 female patients were postmenopausal. The median time between fracture and lab storage was 8 days (interquartile range 9 days) and between fracture and DXA-Scan was 39 days (interquartile range 32 days). Osteoporosis was diagnosed in 46 patients (25.7%) of the total patient group. In all cases of osteoporosis, anti-osteoporosis treatment was started after the DXA-Scan was performed and blood samples were collected. Vitamin D deficiency was found in 34 patients (19.0%). Most fractures were located in the forearm (56.4%) or in the ankle (14.9%; Table 1).

**Table 1:** Anatomical location of fractures in 179 patients

Location, *n* (%)	Frequency
Humerus	11 (6.1)
Forearm	102 (56.4)
Hand	16 (8.8)
Femur	2 (1.1)
Lower leg	9 (5.0)
Ankle	27 (14.9)
Foot	14 (7.7)
Total	181 (100)

Analysis of the patients’ characteristics (Table 2) showed that osteoporotic patients with a fracture had a lower body mass index (mean BMI 24.5 kg/m2 Vs 27.5 kg/m2; *p* < 0.001), used more frequently vitamin D supplementation (10.9% Vs 2.3%; *p* = 0.03), had a lower mean serum PTH (4.0 pmol/L Vs 5.0 pmol/L; *p* = 0.04) and a higher mean eGFR (64.4 min/ml/1.73m2 Vs 59.6 min/ml/1.73m2; *p* = 0.004) compared to the non-osteoporotic patients with a fracture. Patients with a vitamin D deficiency had more often comorbidity (100% Vs 87.6%; *p* = 0.02), a significantly lower mean serum corrected calcium concentration (2.33 mmol/L Vs 2.37 mmol/L; *p* = 0.03) and higher mean serum PTH (6.4 pmol/L Vs 4.4 pmol/L; *p* < 0.001).

**Table 2:** Characteristics of fracture patients, by presence or absence of osteoporosis as diagnosed by endocrinologist, and vitamin D deficiency

Parameter	Osteoporosis			Vitamin D status		
	Osteoporosis (*n* = 46)	No Osteoporosis (*n* = 133)	*p* value	Vitamin D < 30 nmol/L (*n* = 34)	Vitamin D ≥ 30 nmol/L (*n* = 145)	*p* value
General characteristics						
Male gender, *n* (%)	8 (17.4)	32 (24.1)	0.35	9 (26.5)	31 (21.4)	0.521
Age (years), mean (SD)	66.9 (10.3)	64.4 (10.5)	0.17	69.0 (14.6)	64.2 (9.1)	**0.02**
BMI (kg/m2), mean (SD)	24.5 (3.0)	27.5 (4.4)	** < 0.01**	27.5 (4.8)	26.5 (4.2)	0.22
Medical history, *n* (%)	43 (93.9)	118 (88.7)	0.57	34 (100.0)	127 (87.6)	**0.03**
Hypertension	20 (43.5)	44 (33.1)	0.21	17 (50.0)	98 (67.6)	0.05
Diabetes	3 (6.5)	8 (6.0)	0.90	2 (5.9)	9 (6.2)	1.00
COPD	1 (2.2)	6 (4.5)	0.68	1 (2.9)	4 (2.8)	1.00
Hyper(para)thyroidism	1 (2.0)	4 (3.0)	0.77	3 (8.8)	4 (2.8)	0.13
Renal insufficiency	0 (0)	6 (4.5)	0.34	2 (5.9)	4 (2.8)	0.32
≥ 2 previous fractures	13 (28.3)	21 (15.8)	0.06	6 (17.6)	28 (19.3)	1.00
Use of medication, *n* (%)	33 (71.7)	95 (71.4)	0.97	26 (76.5)	102 (70.3)	0.48
Vitamin D	5 (10.9)	3 (2.3)	**0.03**	0 (0.0)	8 (5.5)	0.36
Corticosteroids	2 (4.3)	15 (10.5)	0.37	5 (14.7)	11 (7.6)	0.19
Laboratory results, mean (SD)						
Vitamin D (nmol/L)	61.9 (30.6)	54.4 (27.1)	0.12	–	–	–
Corrected calcium (mmol/L)	2.35 (0.08)	2.37 (0.10)	0.22	2.33 (0.06)	2.37 (0.10)	**0.03**
PTH (pmol/L)	4.0 (1.6)	5.0 (3.3)	**0.04**	6.4 (4.2)	4.4 (2.4)	** < 0.001**
eGFR (min/ml/1.73m2)	64.4 (12.9)	59.6 (8.0)	**0.004**	58.5 (12.9)	61.4 (8.7)	0.12
Sclerostin (pmol/L)	41.9 (14.4)	48.1 (17.5)	**0.03**	42.7 (16.1)	47.4 (17.1)	0.15

Bold values indicate significant *p* values

*BMI* body mass index, *COPD* chronic obstructive pulmonary disease, *PTH* parathyroid hormone, *eGFR* estimated glomerular filtration rate

In the subgroup of 139 women with a mean age of 65.4 years (SD 10.4), osteoporosis was diagnosed in 38 patients (27.4%) and vitamin D deficiency was diagnosed in 25 patients (18.0%). Female osteoporotic patients with a fracture were found to have a lower body mass index (mean BMI 24.3 kg/m2 Vs 27.3 kg/m2; *p* < 0.001), used more frequently vitamin D supplementation (10.5% Vs 2.0%; *p* = 0.047), had a lower mean serum PTH (4.0 pmol/L Vs 5.2 pmol/L; *p* = 0.009) and a higher mean eGFR (64.5 min/ml Vs 59.4 min/ml/1.73m2; *p* = 0.04) compared to the non-osteoporotic patients with a fracture. Female patients with a vitamin D deficiency had a higher BMI (mean BMI 28.2 kg/m2 Vs 26.1 kg/m2; *p* = 0.03), had more often hypertension (56.0% Vs 28.1%; *p* = 0.007), a significantly lower mean serum concentration corrected calcium (2.34 mmol/L Vs 2.38 mmol/L; *p* = 0.04) and higher mean serum PTH (6.6 pmol/L Vs 4.5 pmol/L; *p* = 0.04).

### Sclerostin

**Fig. 1:**
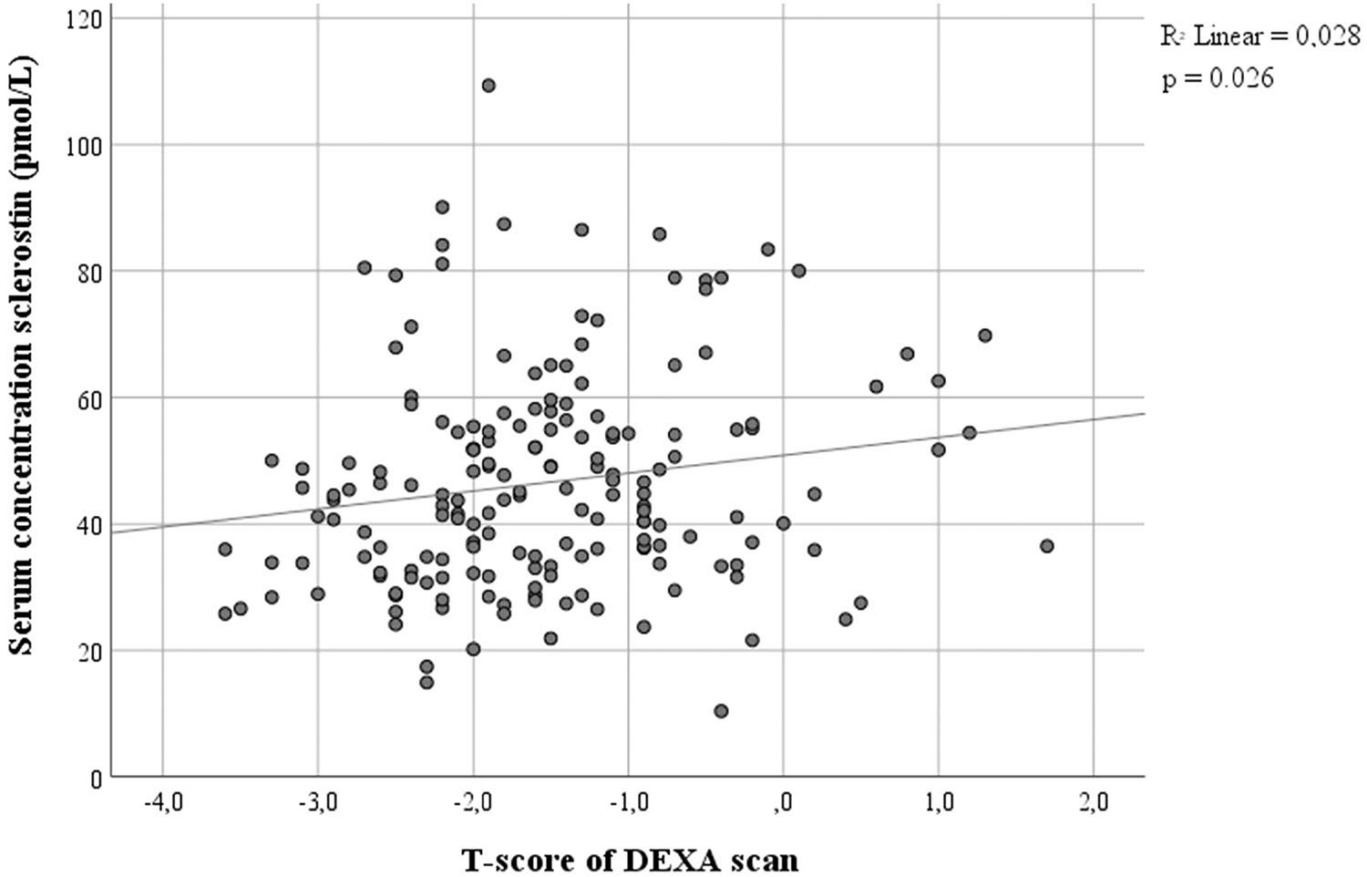
Scatterplot T-score Dexa-scan and serum concentration sclerostin

**Fig. 2:**
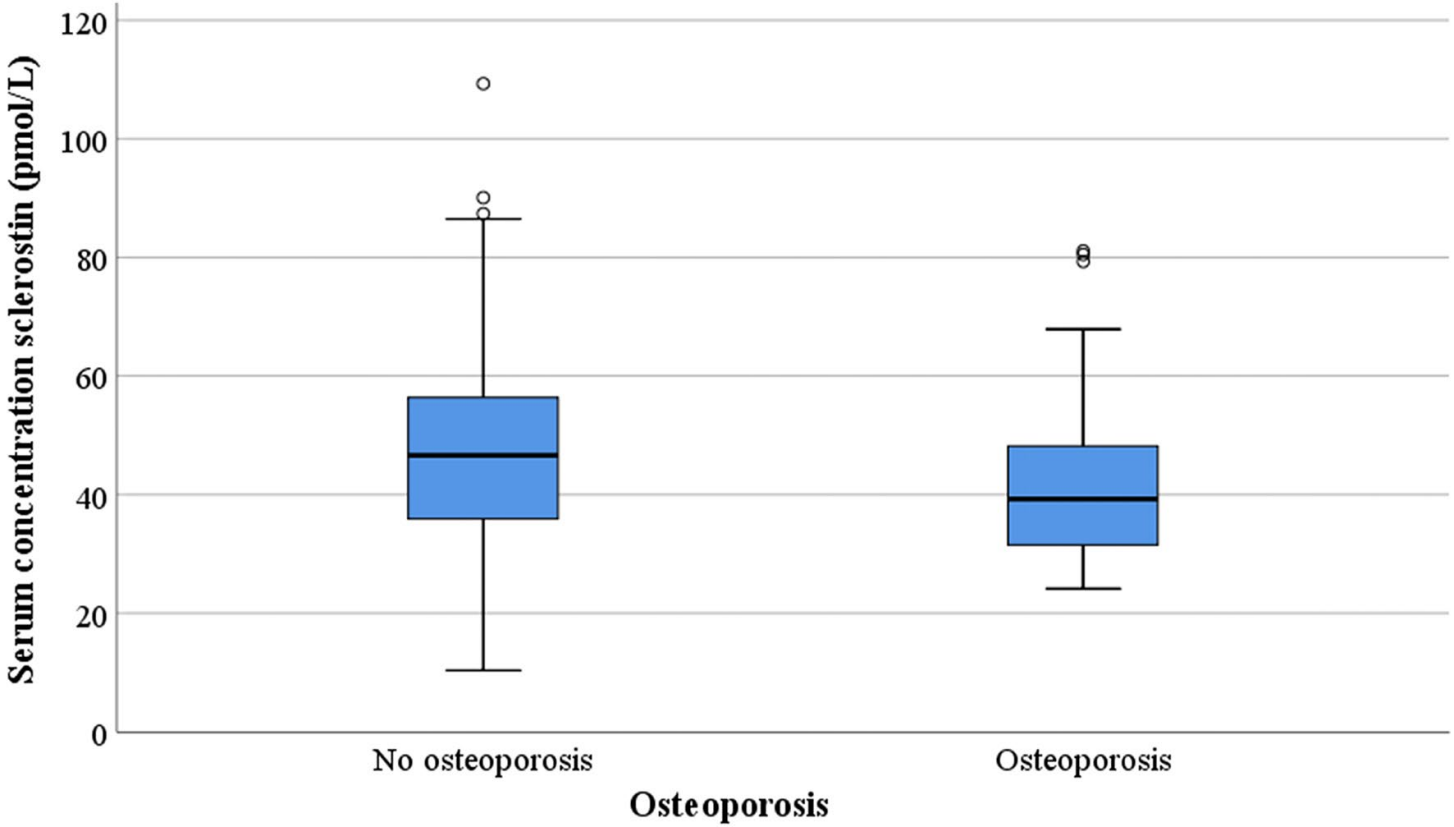
Boxplot of presence or absence of osteoporosis and serum concentration sclerostin

Overall, the study population had a mean serum sclerostin concentration of 46.5 pmol/L (SD 16.9).The bone mineral density was positively associated with the sclerostin levels (*r* = 0.17, *p* = 0.026) (Fig. 1). Subjects with osteoporosis had a significantly lower mean serum sclerostin concentration compared to non-osteoporotic fracture patients (41.9 pmol/L SD 14.4 vs 48.1 pmol/L SD 17.5; *p* = 0.03; Table 2; Fig. 2). After correction for potential confounders (*p* < 0.10 in Table 2) in the multivariate linear regression analysis the difference in sclerostin levels between the groups remained statistically significant (mean difference 6.5 pmol/L, 95% confidence interval 0.4–12.6, *p* = 0.04).

In the subgroup analysis for female fracture patients, a similar difference in sclerostin levels was found between patients with or without osteoporosis (42.4 pmol/L SD 15.2 Vs 48.5 pmol/L SD 18.3; *p* = 0.05). Also after correction for potential confounders a difference of 7.4 pmol (95% confidence interval 0.4–14.4, *p* = 0.04) was found.

### Vitamin D

**Fig. 3:**
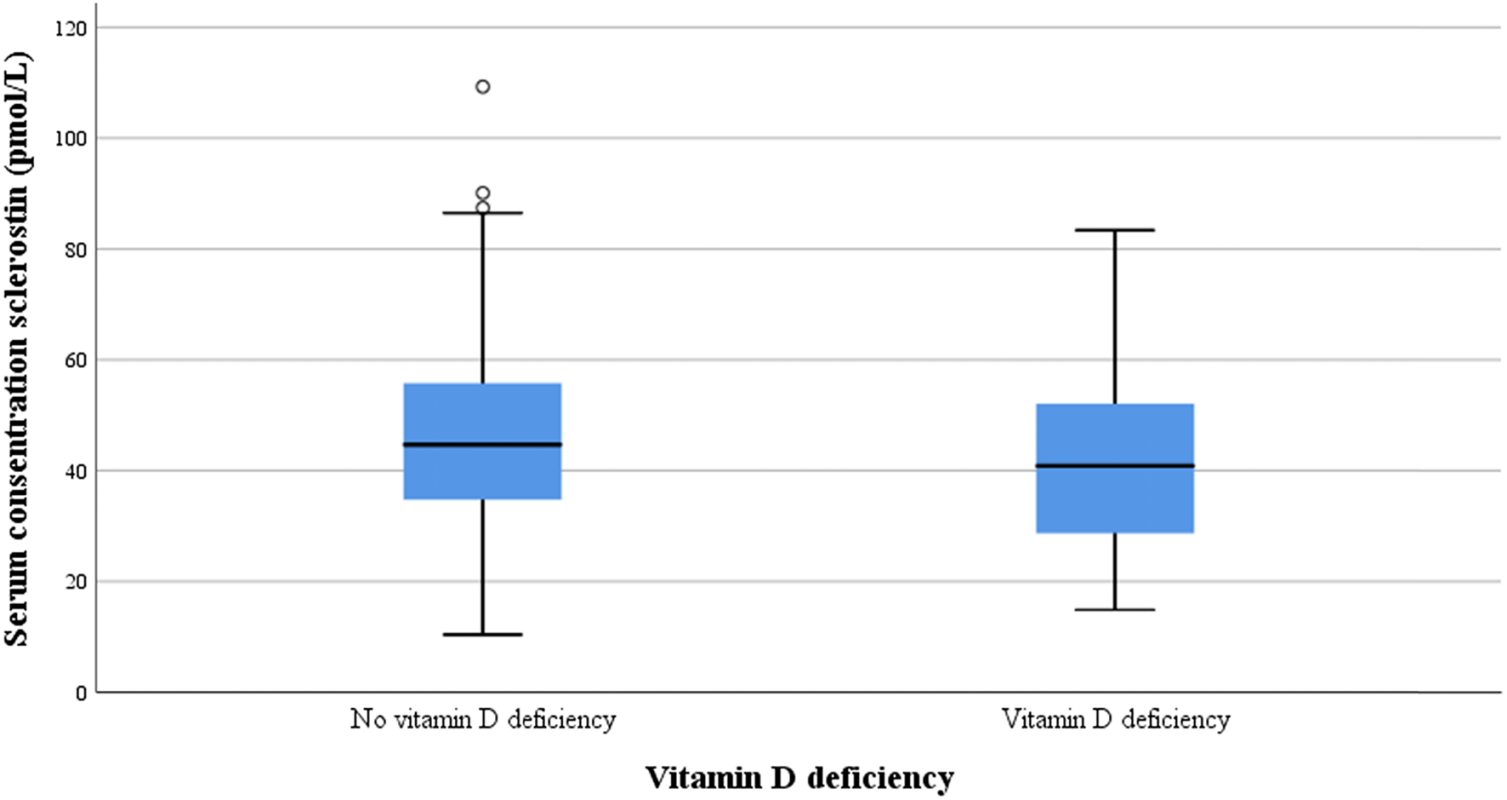
Boxplot of presence or absence of vitamin D deficiency and serum concentration sclerostin

Serum sclerostin levels did not differ between vitamin D-deficient patients and sufficient patients (42.7 pmol/L SD 16.1 vs 47.4 pmol/L SD 17.1; *p* = 0.15; Table 2; Fig. 3), also after correction for potential confounders (mean difference 0.8 pmol/L, 95% confidence interval − 7.4 to 5.8, *p* = 0.81).

Female subjects with vitamin D deficiency showed a significantly lower mean serum concentration sclerostin compared to non-vitamin D-deficient patients(40.5 pmol/L SD 13.4 Vs 48.3 pmol/L SD 18.2; *p* = 0.05). After correction for potential confounders the difference in sclerostin levels was no longer statistically significant (mean difference 3.4 pmol/L, 95% confidence interval − 11.5 to 4.8, *p* = 0.41).

## Discussion

In this study, we found a positive association between bone mineral density and serum sclerostin concentration. We found lower levels of sclerostin in osteoporotic fracture patients with low-energy extremity fractures. No differences in serum levels sclerostin were found with regard to the vitamin D status.

Unlike previously published studies, our study was performed in a general fracture population and did not only investigate the association of serum sclerostin with bone mineral density but also with the diagnosis osteoporosis. Only three studies performed in fracture patients were published previously. They also reported also a positive association with bone mineral density [15, 26, 27]. Both Arasu et al. [26] and Wanby et al. [15] included elderly geriatric patients (mean age 77 and 86, respectively) with hip fractures. Lim et. al [27] included only postmenopausal women (mean age 63) with an osteoporotic fracture (vertebral fracture and non-vertebral fractures in forearm, humerus, and hip). Compared to both Arasu et al. [26] and Wanby et al*.* [15] our study better reflects the mean age of the osteoporotic fracture population and the fractures included were not limited to a single anatomical location. Also, our study population was larger compared to that of Wanby et al. [15]. The study of Lim et al*.* [27] most resembled our study with regard to population size and included types of fractures, although they only included women. Similarly, in our subanalysis including only women, we also found a positive association between lower levels of sclerostin and osteoporotic women.

In various populations, also found a positive association between bone mineral density and serum sclerostin [11–20]. With regard to serum sclerostin levels in osteoporotic versus non-osteoporotic patients, literature is less consistent. Three previous studies have also shown a lower serum sclerostin concentration in patients with osteoporosis compared to non-osteoporotic patients [28, 29, 31]. Lapauw et al*.* [28] compared 116 male idiopathic osteoporosis patients with that of 116 male, and age-matched controls. Basir et al*.* [29] studied 78 renal transplantation patients and Tian et al. [31] studied 500 postmenopausal women. Compared to our study, these studies were performed in non-fracture patients and in relatively young (mean age of 45, 41and 58, respectively) osteoporotic patients. On the other hand, no difference in sclerostin levels between osteoporotic patients and non-osteoporotic patients with spinal cord injury was found [30] and another study found an even higher level of sclerostin among postmenopausal women with osteoporosis compared to non-osteoporotic women [32].

Sclerostin has been reviewed as a potential biomarker for osteoporosis by Ramli et al*.* [25]. Based on the positive association between sclerostin levels and BMD, they concluded that sclerostin might be used as a predictor of osteoporosis but should not replace the DEXA for diagnosing osteoporosis [25]. Also Nagy et al. [48]*.* found sclerostin suitable as a biomarker for osteoporosis. With regard to predicting osteoporotic fractures, Ramli et al*.* [25] found sclerostin not suitable due to heterogeneous results found in their review [25]. Another review by Kim, et al. [49] also found ambiguous results and considered sclerostin not a predictor of a fracture.

Since sclerostin is predominantly synthesized by osteocytes, a possible explanation of the lower levels of sclerostin in osteoporotic patients is the age-dependent reduction in osteocyte number/density [50–52] and change in morphology [53]. These alteration might result in an decreased functioning of the osteocyte, including the production of sclerostin. Moreover, in two studies, a decreased osteocyte density was found in patients with osteoporosis [54, 55]. In osteoporosis, the occurrence and density of mineralized lacunae is higher. This lacunar mineralization is associated with osteocyte apoptosis [56]. The involvement of osteocytes in osteoporosis is known from disuse osteoporosis; bone loss due to local skeletal unloading or systemic immobilization which results in increased osteocyte apoptosis and increased local sclerostin secretion by osteocytes with subsequent relative increased bone resorption compared to decreased bone formation [2]. Another explanation may be the presence of a reversed association between sclerostin and inferior bone quality. A negative feedback loop on sclerostin may exist, which is activated in osteoporosis or in other conditions causing a worse bone mineral density. Due to a lower concentration of sclerostin, less inhibition of bone formation and less stimulation of bone resorption occur which prevents further deterioration of bone mineral density. Also, the occurrence of the fracture itself could be considered as a bias influencing the amount of sclerostin, since sclerostin seems to be involved in fracture healing besides osteocytes [4–6, 8]. Literature on the effect of a fracture on the levels of sclerostin is scarce. Sarahrudi et al. [4] and Arasu et al*.* [26] reported an increased serum concentration in fracture patients compared to non-fracture patients. However, Wanby et al. [15] and Dovjak et al*.* [57] found no difference in sclerostin levels between fracture and non-fracture patients. Lim et al. [27] even found a lower level of sclerostin in fracture patients.

Our results did not show a difference in sclerostin levels between vitamin D-deficient patients and patients without a deficiency. Based on literature, it may be assumed that sclerostin is involved in the process of bone mineralization because of its potential interaction with vitamin D, PTH and FGF-23 [3, 43]. The exact mechanism of this regulation remains unknown especially due to the different results of in vitro and in vivo studies [3].

A limitation of this study was that seasonal variation in sclerostin was not accounted for, even though Dawson-Hughes et al. [58]. showed that this type of variation exists. We do, however, expect this to be of little influence since patients were included year-round. Furthermore, this study featured a population with a wide array of fractures leading to heterogeneity in the study population. The results are, therefore, not applicable to one specific fracture type or patient group. Another limitation is posed by the influence of the type of assay used for determination of serum sclerostin levels. Durosier et al*.* [16] tested multiple sclerostin assays in the same population and found that the MSD assay used in this study seems only to detect intact SOST molecules, causing an underestimation of the total serum concentration of sclerostin. This may hamper the comparison between levels of sclerostin found in our study and those in other studies, but not within our data, since in our study, the same method was used for all samples. Although this study had a retrospective design, the data were obtained with a standardized follow protocol via our fracture liaison service.

All patients were seen and investigated via the same fracture liaison service. However, the moment of blood investigations and biobanking differenced between the patients selected from the Biobank for Bone and Mineral Disorders and the vitamin D-study biobank. In case of the vitamin D study, laboratory investigations as well as the biobank storage were performed during the first follow-up visit at the outpatient clinic. In the other patients, laboratory investigations as well as the biobank storage were performed during the fracture liason service. All Dexa-scans were planned and performed via the fracture liaison service, this explains the time range in BMD measurements as not all measurements can be performed at short notice or due to a (patient or hospital) delay in the appointment at the FLS service. Overall, data from the fracture liaison service are based on daily practise and might induce heterogeneity. Also, the different timing of blood biobanking via two protocols contributes to this heterogeneity.

In conclusion, the bone mineral density in patients with an extremity fracture was positively associated with sclerostin levels and osteoporotic fracture patients had significantly lower levels of sclerostin compared to non-osteoporotic fracture patients. No correlation was found between serum vitamin D levels and sclerostin levels. Future research should focus on the use of sclerostin as biomarker for osteoporosis in fracture patients.

## Data Availability

Not applicable.
